# A pathological complete response after neoadjuvant triplet chemotherapy for locally advanced transverse colon cancer

**DOI:** 10.1016/j.ijscr.2020.05.077

**Published:** 2020-06-06

**Authors:** Tetsuro Tominaga, Takashi Nonaka, Kazuhiro Tabata, Terumitsu Sawai, Takeshi Nagayasu

**Affiliations:** a Departments of Surgical Oncology, Nagasaki University Graduate School of Biomedical Science, 1-7-1 Sakamoto, Nagasaki, 852-8501, Japan; b Departments of Pathology, Nagasaki University Graduate School of Biomedical Science, 1-7-1 Sakamoto, Nagasaki, 852-8501, Japan; c Departments of Cardiopulmonary Rehabilitation Science, Nagasaki University Graduate School of Biomedical Science, 1-7-1 Sakamoto, Nagasaki, 852-8501, Japan

**Keywords:** CRC, colorectal cancer, LACC, locally advanced colon cancer, NAC, neoadjuvant chemotherapy, pCR, pathological complete response, Locally advanced colon cancer, FOLFOXIRI, Complete response

## Abstract

**Introduction:**

Perioperative chemotherapy could improve oncological outcomes for patients with advanced colon cancer. However, the effectiveness of triplet chemotherapy in the neoadjuvant setting is still unknown.

**Presentation of case:**

A 61-year-old man was referred to our hospital due to abdominal distention. Abdominal computed tomography showed a huge, 18-cm mass in the right upper abdomen. Biopsy showed well-differentiated adenocarcinoma. Locally advanced transverse colon cancer T4b N2a M0 Stage IIIC was diagnosed. Considering the extensive invasion to surrounding organs and difficulties in achieving margin-negative surgery, an emergency ileostomy was performed first. Then, neoadjuvant chemotherapy (NAC) consisting of a combination of 5-fluorouracil (5-FU), oxaliplatin, irinotecan, and leucovorin (FOLFOXIRI) was planned, followed by primary tumor resection. After 6 courses of treatment, the primary tumor shrank remarkably. Finally, laparoscopic radical extended right hemi-colectomy was performed. There were no residual tumor cells in resected specimens, including the primary tumor and surrounding lymph nodes. The pathological diagnosis was complete response.

**Conclusion:**

A case of pathological complete response after neoadjuvant treatment followed by radical resection was reported. Further research is needed to confirm the appropriate indications for neoadjuvant FOLFOXIRI therapy for patients with LACC.

## Highlights

• Perioperative chemotherapy could improve oncological outcome for colon cancer.
• The effectiveness of triplet chemotherapy as neoadjuvant setting is still unknown.
• A pathological complete response after neoadjuvant triplet chemotherapy was described.

## 1 Introduction

Colorectal cancer (CRC) is the 3rd commonest diagnosed cancer, and it is estimated that over 130,000 patients were diagnosed with CRC worldwide [1]. Locally advanced colon cancer (LACC) is defined as primary cancer with invasion to adjacent organs or extensive lymph node involvement. The standard care in LACC is complete surgical resection followed by adjuvant chemotherapy [2]. However, patients with LACC have a low rate of complete resection and a high incidence of morbidity after colorectal surgery because of the required multi-visceral resection [3]. The development of postoperative complications is one of the reasons for a longer hospital stay [4], which could delay adjuvant chemotherapy. Furthermore, treatment delay has been found to correlate significantly with a poor prognosis [5].

Recently, laparoscopic surgery has become the standard approach for colon cancer, with better short-term outcomes including less blood loss, quicker recovery of bowel function, and less postoperative complications [6]. However, we have often had to perform open surgery for patients with LACC because of a huge primary tumor with bowel obstruction that prevented an adequate operative field with the laparoscopic approach.

Neoadjuvant treatment is expected to have advantages such as tumor regression, tumor shrinkage, and down staging, making successful laparoscopic surgery more likely [7]. Furthermore, such treatment could avoid delaying chemotherapy due to surgery-related complications that would affect the start of adjuvant chemotherapy [8]. A randomized, controlled study assessing the effect of preoperative chemotherapy using FOLFOX or CapeOx for LACC patients showed that preoperative chemotherapy resulted in significant downstaging compared with postoperative therapy [9]. However, there are few reports examining the effectiveness of triplet chemotherapy, including FOLFOXIRI, in the NAC setting for patients with LACC [10].

A case of pathological complete response (pCR) of locally advanced transverse colon cancer after preoperative FOLFIXIRI therapy followed by laparoscopic right hemi-colectomy is reported.

This work is reported in line with the SCARE criteria [11].

## 2 Case presentation

**Fig. 1:**
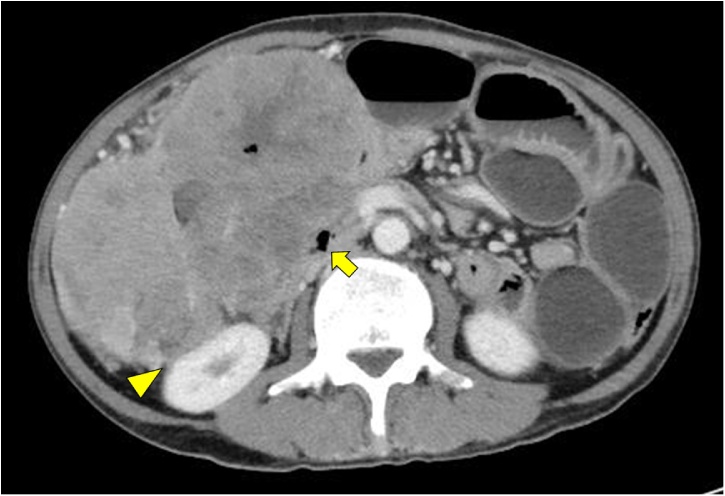
Abdominal CT on admission. Abdominal CT shows a huge, 18-cm mass in the right upper abdomen (Fig. 1). The tumor is located very close to surrounding organs such as the duodenum (arrow) and right kidney (arrow head).

**Fig. 2:**
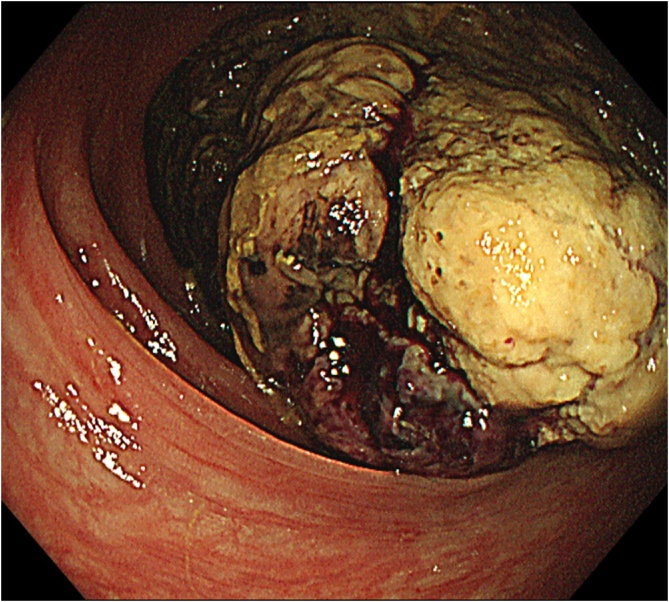
Colonoscopy findings. Colonoscopy shows a circumferential type 2 tumor at the transverse colon. The scope cannot pass through to the oral side of the tumor.

**Fig. 3:**
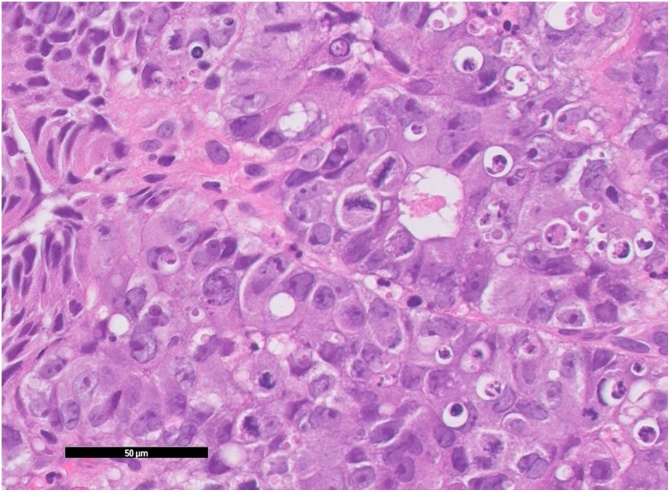
Histology of the biopsy specimen. HE stains show poorly differentiated adenocarcinoma with atypical epithelial cells that have proliferated solidly with focal glandular structure, and many apoptotic cells and mitotic figures.

**Fig. 4:**
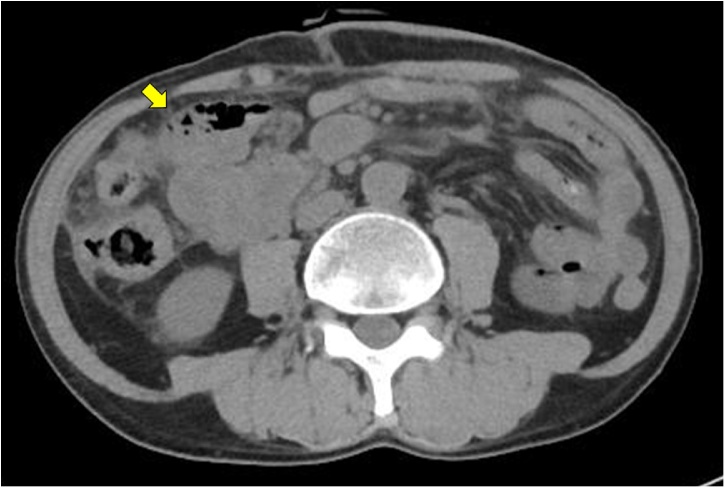
Abdominal CT after 6 courses of neoadjuvant chemotherapy. After 6 courses of treatment, the primary tumor has shrunk remarkably to 5.0 cm. Invasion to surrounding organs is not observed.

**Fig. 5:**
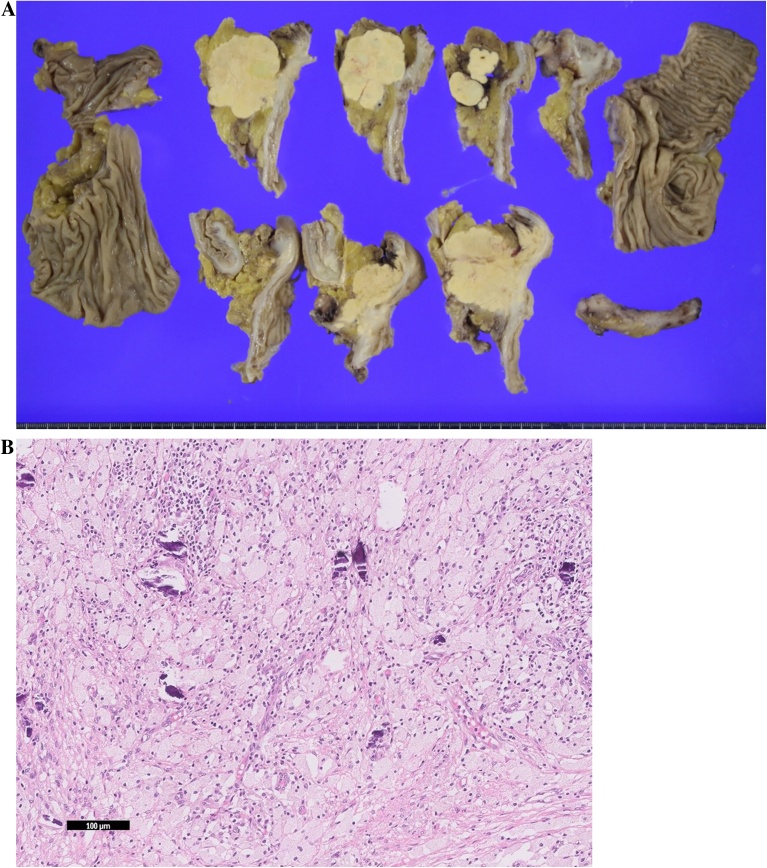
Gross appearance of the cut surface. The yellow-whitish area indicates degenerative change of the carcinoma due to neoadjuvant chemotherapy (a). Histology of the degenerative cancer area. Foamy macrophages and psammomatous bodies without viable cancer cells are seen (b).

A 61-year-old man was referred to our hospital due to abdominal distention. Physical examination demonstrated a huge, hard, tender mass in the right upper quadrant of the abdomen. Laboratory data showed inflammation (white blood cells, 15,600/μL; C-reactive protein, 19.1 mg/L), anemia (hemoglobin, 9.8 g/dL), malnutrition (albumin 2.0 g/dL), and an elevated tumor marker (carcinoembryonic antigen 64.1 U/mL). Abdominal computed tomography showed a huge 18-cm mass in the right upper abdomen (Fig. 1). The tumor was located close to surrounding organs such as the duodenum and right kidney. Enlarged surrounding lymph nodes were also noted. There were no distant metastases. The small intestine was dilated due to obstruction by primary tumor. Colonoscopy showed a circumferential type 2 tumor located at the transverse colon (Fig. 2). The scope could not pass through to the oral side of the tumor. Biopsy showed poorly differentiated adenocarcinoma (Fig. 3); RAS status was mutant. Though an ileus tube was inserted, the patient’s symptoms did not improve. Finally, locally advanced transverse colon cancer T4b N2a M0 Stage IIIC was diagnosed. Considering the extensive invasion to surrounding organs and difficulties in achieving margin-negative surgery, emergency ileostomy was performed first. Neoadjuvant chemotherapy (NAC) consisting of a combination of 5-fluorouracil, oxaliplatin, irinotecan, and leucovorin (FOLFOXIRI) plus bevacizumab, followed by primary tumor resection, was then planned. During NAC, the patient developed grade 2 neutropenia, but the planned course of treatment was completed. After 6 courses of treatment, the primary tumor had shrunk remarkably, from 18.0 cm to 5.0 cm (Fig. 4), and invasion to surrounding organs was not observed. Laparoscopic extended right hemi-colectomy was then performed. First, the ileostomy was closed. Then, EZ access (Hakko-medical, Tokyo, Japan) was inserted through the wound. Five ports, one for the scope and the others for the handling forceps, were used. The procedure was started with the cranial approach for right colectomy, beginning with hepato-colic ligament resection. From the cranial view, the duodenum was not invaded by the primary tumor. After resection of the hepato-colic flexure, the pedicle of the ileocecal artery and vein was grasped by the assistant’s forceps. Then, the regional lymph node and vessels were resected. To mobilize the intestine, the mesentery proper was grasped, and the insertion of the mesentery proper was cut. The tumor had not invaded the right kidney. To remove the lesion from the body, the wound was dilated to 5 cm. The tumor was then resected by a suture instrument. A functional end-to-end anastomosis was created. Macroscopic examination showed a 4.8cm × 4.1cm tumor at the transverse colon (Fig. 5a). Histopathologically, the primary tumor and enlarged lymph nodes consisted of fibrous or granuloma-like tissues, and no residual cancer cells were found (pCR) (Fig. 5b). The final diagnosis was transverse colon cancer, ypT0 ypN0 ypStage 0. The patient’s postoperative course was uneventful, and he was discharged from our hospital on postoperative day 10.

## 3 Discussion

In the present case report, a pCR was achieved after neoadjuvant FOLFOXIRI plus bevacizumab for LACC.

Several guidelines recommend wide surgical resection as the treatment strategy for LACC [2,12]. Following surgery, the standard treatment is doublet chemotherapy with oxaliplatin and a fluoropyrimidine as adjuvant chemotherapy. In previous reports, it was suggested that adjuvant chemotherapy should be started within 8 weeks after surgery. Delaying the initiation of adjuvant treatment was significantly correlated with a poor prognosis [13,14]. Patients with LACC sometimes required multivisceral resection to maintain an adequate resection margin. In addition, multivisceral resection was reported to correlate with a high incidence of postoperative complications, which could cause a longer hospital stay and delay the start of systemic chemotherapy [15]. From the 2016 version of the NCCN guidelines, the panel added NAC as a treatment option for clinical T4b patients [16]. NAC has some potential benefits, including a better compliance rate, tumor shrinkage, and downstaging [7]. Then, surgery after NAC could resect the tumor more completely and radically. In the present case, abdominal CT on admission showed a huge tumor located close to the duodenum and right kidney, and combined resection of these organs would have been needed for radical resection. However, after 6 courses of FOLFOXIRI plus bevacizumab, the main tumor shrank from 18 cm to 5 cm, and radical resection could be performed without multivisceral resection.

Some RCTs reported that laparoscopic surgery had less blood loss, shorter time to pass first flatus, less use of analgesics after surgery, and a shorter hospital stay [6,17]. For the present patient, open surgery was originally planned due to concerns about an inadequate operation field due to the huge tumor with bowel obstruction. However, laparoscopic surgery was ultimately safely completed because of shrinkage of the main tumor and surrounding lymph nodes, which could have resulted in the better recovery after surgery.

The major potential drawback of NAC is that disease progression during NAC may preclude curative surgery. In regard to LACC patients, several studies have evaluated the effect of NAC, and they reported that NAC is safe and feasible [7,9,18,19]. Arredondo and colleagues assessed the radiological and pathological findings induced by NAC with oxaliplatin and fluoropyrimidine-based chemotherapy for LACC. After NAC, significant tumor volume reduction of 62 % was achieved on CT, and no progressive disease was reported during treatment [19]. Recently, the FOxTROT trial, which aimed to investigate the feasibility, safety, and efficacy of NAC for colon cancer, was reported [9]. About 90 % of the patients completed planned chemotherapy, and all tumors were resected without an increase in postoperative morbidities. No patients receiving NAC experienced up-staging after surgery.

In the FOxTROT trial, the patients underwent 3 courses (six weeks) of NAC followed by surgery [9]. In the present case, complete R0 surgery would have been difficult unless combined resection of the surrounding organs were performed. Therefore, stronger triplet (FOLFOXIRI) therapy was selected to obtain more tumor shrinkage. After six courses (12 weeks) of therapy, the tumor could be resected completely without multivisceral resection because it had shrunk remarkably. The appropriate timing of surgery was then considered, and the tumor could finally be resected safely and completely.

However, the appropriate timing of surgery during triplet chemotherapy is not well known. A further large-scale study is needed to clarify this issue.

There is no consensus about which regimen, doublet or triplet, should be selected for NAC. Several clinical trials selected doublet therapy using oxaliplatin and fluoropyrimidine as NAC for LACC patients, and overall response rates ranged from 13.6% to 30.8%, and pCR ranged from 0 % to 6.8 % [7,9,18,19]. Few studies have assessed the effectiveness of triplet therapy using 5-fluorouracil, oxaliplatin, and irinotecan in the NAC setting for LACC [10]. Preoperative therapy resulted in significant down staging compared to the clinical stage. The overall response rate was 82.6 %, including 4.3 % with pCR. A previous report showed that pCR after chemo(radio)therapy is associated with a greatly improved cancer prognosis in locally advanced cancer [20,21]. In the present case, the primary tumor was huge, 18 cm in size, and triplet therapy was selected as NAC, hoping for downsizing and early improvement of clinical symptoms. Finally, the tumor shrank remarkably, and it was possible to perform radical right hemicolectomy laparoscopically and achieve pCR pathologically.

On the other hand, severe adverse events including neutropenia, diarrhea, and nausea occurred significantly more often in patients receiving triplet therapy compared to doublet therapy [22,23]. In the present case, the patient developed grade 2 neutropenia after 4 courses of therapy, but he completed planned treatment.

The best method of follow-up for colon cancer patients with pCR after neoadjuvant treatment is controversial. In rectal cancer, distant metastasis still remains a concern in pCR patients after chemoradiotherapy [24]. Therefore, adjuvant chemotherapy is recommended for selected patients. A recent meta-analysis also showed that rectal cancer patients with pCR after chemoradiotherapy who received adjuvant chemotherapy showed significantly improved overall survival [25]. In the present case, the patient received six courses of adjuvant chemotherapy, and there was no evidence of recurrence or metastasis.

## 4 Conclusion

A case of pCR after neoadjuvant treatment followed by radical resection was reported. Further research is needed to confirm the appropriate indications for neoadjuvant FOLFOXIRI therapy for patients with LACC.

## Conflicts of interest

None

## Sources of funding

None

## Ethical approval

Ethical approval has been exempted by our institution

## Consent

Written informed consent was obtained from the patient for publication of this case report and accompanying images. A copy of the written consent is available for review by the Editor-in-Chief of this journal on request

## Author contribution

Tetsuro Tominaga and Takashi Nonaka conceptualized the study. Kazuhiro Tabata made a pathological diagnosis.Terumitsu Sawai and Takeshi Nagayasu provided input on the manuscript

## Registration of research studies

Researchregistry1025

## Guarantor

Tetsuro Tominaga

Provenance and peer review

Not commissioned, externally peer-reviewed
